# LETM1: A Single Entity With Diverse Impact on Mitochondrial Metabolism and Cellular Signaling

**DOI:** 10.3389/fphys.2021.637852

**Published:** 2021-03-18

**Authors:** Gayathri K. Natarajan, Jyotsna Mishra, Amadou K. S. Camara, Wai-Meng Kwok

**Affiliations:** ^1^Department of Anesthesiology, Medical College of Wisconsin, Milwaukee, WI, United States; ^2^Department of Physiology, Medical College of Wisconsin, Milwaukee, WI, United States; ^3^Cancer Center, Medical College of Wisconsin, Milwaukee, WI, United States; ^4^Cardiovascular Center, Medical College of Wisconsin, Milwaukee, WI, United States; ^5^Department of Pharmacology and Toxicology, Medical College of Wisconsin, Milwaukee, WI, United States

**Keywords:** leucine-zipper EF-hand containing transmembrane 1, mitochondrial calcium hydrogen exchanger, mitochondrial potassium hydrogen exchanger, mitochondrial calcium handling, carboxy-terminal-modulator-protein, Wolf-Hirschhorn syndrome, cancer biology, cell metabolism, bioenergetics

## Abstract

Nearly 2 decades since its discovery as one of the genes responsible for the Wolf-Hirschhorn Syndrome (WHS), the primary function of the leucine-zipper EF-hand containing transmembrane 1 (LETM1) protein in the inner mitochondrial membrane (IMM) or the mechanism by which it regulates mitochondrial Ca^2+^ handling is unresolved. Meanwhile, LETM1 has been associated with the regulation of fundamental cellular processes, such as development, cellular respiration and metabolism, and apoptosis. This mini-review summarizes the diversity of cellular functions impacted by LETM1 and highlights the multiple roles of LETM1 in health and disease.

## Introduction

Mitochondria accumulate large amounts of Ca^2+^ *via* highly regulated mechanisms of Ca^2+^ transport across the mitochondrial membranes and Ca^2+^ handling in the matrix (Rizzuto et al., 2012) and thereby, attenuate deleterious increases in cytosolic Ca^2+^ (Hajnóczky et al., 2014). Ca^2+^ uptake into mitochondria by the mitochondrial Ca^2+^ uniporter (MCU) macromolecular complex (MCUC) and Ca^2+^ efflux through the electrogenic Na^+^/Li^+^/Ca^2+^ exchanger (NCLX) are among the well-studied mechanisms of mitochondrial Ca^2+^ transport (Palty et al., 2010; Blomeyer et al., 2013; Mishra et al., 2017). Recently, the Leucine zipper EF-hand containing transmembrane 1 (LETM1) protein, expressed in the inner mitochondrial membrane (IMM) has been implicated in mitochondrial Ca^2+^ regulation. However, the mechanism by which LETM1 regulates Ca^2+^ is unresolved and controversial.

The *Letm1* gene was originally identified as one of the genes deleted in patients afflicted with the Wolf-Hirschhorn Syndrome (WHS), a contiguous gene deletion disorder marked by severe growth and intellectual disability, hypotonia, and seizures (Endele et al., 1999; Schlickum et al., 2004). It is evolutionarily conserved in all eukaryotes, plants, and animals (Zhang et al., 2012; Tang et al., 2018; Lin and Stathopulos, 2019) with ubiquitous expression in mammalian tissues (Fagerberg et al., 2014; Uhlén et al., 2015). *Letm1* is characterized as an essential gene, such that haploinsufficiency leads to manifestation of disease phenotype (Endele et al., 1999; Hart et al., 2014), and homozygous deletion is embryonically lethal (McQuibban et al., 2010; Jiang et al., 2013).

**Figure 1:**
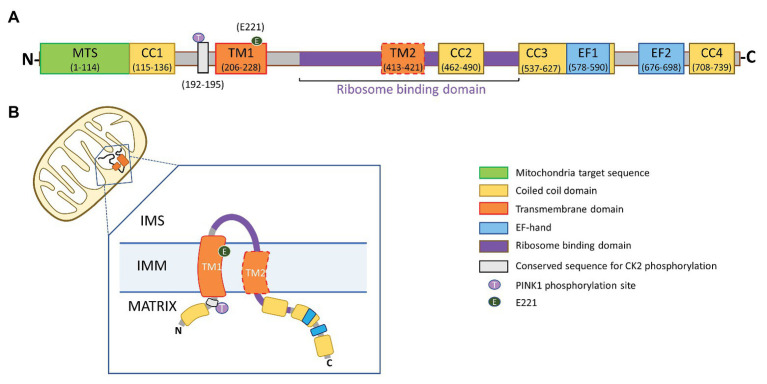
Proposed topology of leucine-zipper EF-hand containing transmembrane 1 (LETM1). **(A)** Domain architecture of LETM1. MTS, mitochondrial targeting sequence; CC1, CC2, CC3, and CC4, coiled-coil domains 1–4; TM1, conserved transmembrane domain 1; TM2, putative transmembrane domain 2; EF1, EF2, non-canonical Ca^2+^-binding EF-hand domains; and Gray Box – TXXR motif, a conserved consensus site for phosphorylation by protein kinase C and casein kinase 2 (CK2). The numbers in parentheses represent amino acid residue for human LETM1 sequence (Uniprot accession number: Q2VYF4). **(B)** Both the N- and C-termini of the LETM1 subunit are proposed to reside in the mitochondrial matrix. A portion of the ribosome binding domain is exposed to the inter-membrane space (IMS).

The LETM1 subunit (83.4 kDa) has a short hydrophobic N-terminal, a conserved proline-rich transmembrane domain, a hydrophilic C-terminal in the matrix, with two to four coiled-coil domains (CCDs), and a characteristic leucine-zipper sequence (Endele et al., 1999). Depending on the species, the C-terminus may include either one or two non-canonical Ca^2+^-binding EF-Hand motifs, or none as in the orthologs from yeast and from the protozoan *Trypanosome brucei* (Nowikovsky et al., 2004; Hasegawa and van der Bliek, 2007). A TXXR motif, a consensus site for phosphorylation by PKC and Casein Kinase 2 (CK2), flanks the N-terminus of the conserved transmembrane domain (Figure 1). Electron microscopy data suggest that LETM1 subunits oligomerize into a putative hexameric configuration to form a functional exchanger with pH-sensitive conformation and conductance properties (Hasegawa and van der Bliek, 2007; Shao et al., 2016). The N-terminus of LETM1 was presumed to face the mitochondrial intermembrane space (IMS); however, recent predictions of a putative second transmembrane domain in the LETM1 protein has led to a reevaluation of the structure and orientation of the LETM1 subunit in the IMM (Lee et al., 2017; Austin and Nowikovsky, 2019).

**Figure 2:**
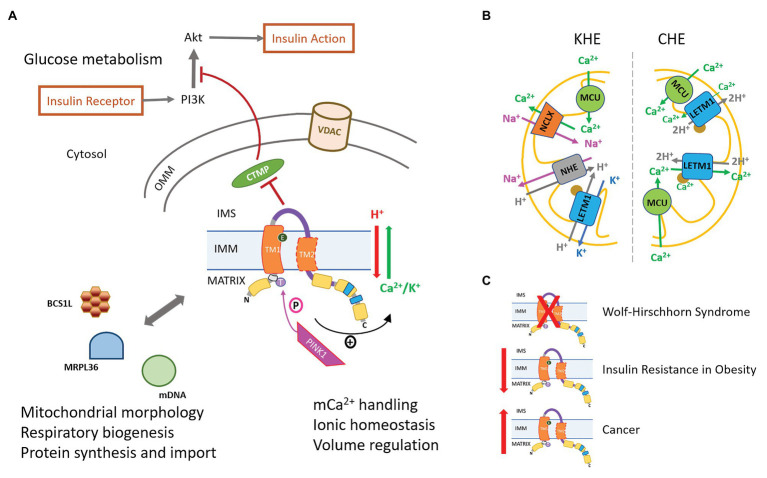
LETM1-mediated regulation of cellular physiology and pathophysiology. **(A)** LETM1-mediated Ca^2+^/H^+^ exchanger (CHE) or K^+^/H^+^ exchanger (KHE) activity regulates mitochondrial Ca^2+^ handling and bioenergetics. The serine-threonine kinase PINK1, associated with Parkinson’s Disease (PD), phosphorylates LETM1 at T192 and enhances exchanger activity (see text for more details). Interactions of LETM1 with signaling proteins, such as CTMP, BCS1L, and MRPL36, regulate glucose metabolism, mitochondrial morphology, and protein assembly in the inner mitochondrial membrane (IMM), respectively. VDAC, voltage-dependent anion channel **(B)** Proposed mechanisms of LETM1 as KHE (left) or CHE (right). The brown circle represents EF-hand in mammalian LETM1. **(C)** Disease phenotypes associated with either the absence [Wolf-Hirschhorn Syndrome (WHS)], decreased expression (Insulin resistance in obesity), or increased expression (cancer) of LETM1.

The functional role of LETM1 in mitochondria is controversial and extensively debated as to whether it is primarily a K^+^/H^+^ exchanger (KHE) as originally surmised or a Ca^2+^/H^+^ exchanger (CHE), as subsequent studies have shown (Nowikovsky et al., 2012; Austin and Nowikovsky, 2019; Li et al., 2019; Lin and Stathopulos, 2019). Regardless, LETM1 is implicated in mitochondrial and cellular functions, such that loss of LETM1 impacts mitochondrial ionic homeostasis, bioenergetics, morphology and biogenesis, cell viability, and development. Furthermore, LETM1 has been shown to interact with proteins that regulate varied signaling processes, such as import and assembly of critical respiratory supercomplexes in the IMM, cellular glucose metabolism, and neuronal function (Figure 2). Consequently, changes in LETM1 expression and function are associated with pathophysiologies, such as insulin resistance in obesity, tumorigenesis, and seizures (Hasegawa and van der Bliek, 2007; Dimmer et al., 2008; Tamai et al., 2008; Jiang et al., 2013; Hart et al., 2014; Park et al., 2014; Li et al., 2019). It is unclear as to how LETM1, localized to the IMM, regulates and impacts diverse cellular functions. It is expected that as a CHE and/or KHE in the IMM, LETM1 would regulate mitochondrial function. However, the studies revealing interactions between LETM1 and regulatory proteins suggest that LETM1 function may extend beyond its exchanger role in the IMM. This mini-review briefly summarizes the current status of the debate regarding LETM1 function as a CHE or KHE. It also highlights the diversity of cellular functions impacted by the loss or gain of LETM1 and the possible mechanisms involved in LETM1-mediated regulation of mitochondrial metabolism and cellular signaling.

## LETM1: Che or Khe?

Early evidence for LETM1 as a putative KHE (LETM1-KHE) came from studies in mitochondria expressing MDM38*Δ*, a mutant yeast ortholog of LETM1. MDM38Δ mitochondria exhibited higher matrix K^+^ content, decreased IMM potential (ΔΨ)_m_, loss of K^+^ acetate-induced swelling, and dissolution of cristae structure and mitochondrial morphology (Nowikovsky et al., 2004; Froschauer et al., 2005). These effects were replicated in mitochondria of other lower and higher organisms and cell cultures with knockout or knockdown of corresponding LETM1 orthologs, including *Caenorhabditis elegans*, HeLa cells, *Drosophila melanogaster*, and *T. brucei* (Hasegawa and van der Bliek, 2007; Dimmer et al., 2008; McQuibban et al., 2010; Hashimi et al., 2013). The K^+^ ionophore, nigericin, or the expression of human LETM1 reversed these effects, suggesting that the swelling and resultant effects on morphology and function were caused by the loss of an evolutionarily conserved KHE function in LETM1-deficient mitochondria (Nowikovsky et al., 2004).

However, in contrast with yeast or the protozoan *T. brucei*, LETM1 orthologs of higher organisms possess EF-hands, which may act as Ca^2+^ sensors and implicate LETM1 in Ca^2+^ regulation. Accordingly, LETM1 was identified as a putative CHE (LETM1-CHE) in a genome-wide RNAi screening in *D. melanogaster* cells, where mitochondrial Ca^2+^ influx/efflux was coupled to H^+^ extrusion/influx, respectively. LETM1-knockdown (LETM1-KD) decoupled the Ca^2+^/H^+^ antiport while LETM1 overexpression increased pH-induced Ca^2+^ transport 5-fold (Jiang et al., 2009). In subsequent studies, recombinant LETM1 reconstituted into liposomes exhibited LETM1-mediated Ca^2+^ transport that is bidirectional and insensitive to K^+^ and Na^2+^, indicating that LETM1 is a CHE (Jiang et al., 2009; Tsai et al., 2014; Shao et al., 2016). Both Ca^2+^ uptake and release were driven by changes in pH (Jiang et al., 2009; Tsai et al., 2014) and enhanced by the phosphomimetic T192E located in the N-terminus of the LETM1 subunit (Huang et al., 2017b).

In cells and isolated mitochondria, it is unclear whether LETM1-CHE mediates either mitochondrial Ca^2+^ uptake or efflux. In histamine-stimulated HeLa cells, LETM1-KD or a mutation of negative-charged glutamate residue in the transmembrane domain increased mitochondrial Ca^2+^, indicating a loss of LETM1-mediated Ca^2+^ efflux (Shao et al., 2016). In isolated rat heart mitochondria, Na^+^-independent Ca^2+^ efflux increased with extra-matrix acidification and with increasing Ca^2+^ load and LETM1-KD in permeabilized H9c2 cells modified Ca^2+^ efflux, indicating that, in these cells, LETM1-CHE mediates Ca^2+^ efflux (Natarajan et al., 2020). In contrast, in another study using histamine-stimulated HeLa cells, LETM1-KD significantly diminished Ca^2+^ uptake in the submicromolar Ca^2+^ range (Jiang et al., 2009). In endothelial cells, LETM1, along with MCU, may mediate mitochondrial Ca^2+^ uptake in response to slow and small Ca^2+^ elevations due to store-operated Ca^2+^ entry (SOCE) following Ca^2+^ depletion in the sarco/endo-plasmic reticulum (SR/ER) and/or failure of Ca^2+^ sequestration through the sarco/endo-plasmic reticular Ca^2+^ ATPase (SERCA; Waldeck-Weiermair et al., 2013). In this study, LETM1-KD significantly diminished mitochondrial Ca^2+^ uptake under conditions of slow Ca^2+^ increases during SOCE (Patergnani et al., 2011; Waldeck-Weiermair et al., 2011, 2013). Other studies have shown that LETM1-CHE may mediate both Ca^2+^-influx and efflux. For instance, Doonan et al. (2014) showed that in histamine-challenged HeLa cells, LETM1-KD or the expression of a LETM1-mutant with deleted EF-hands significantly diminished both Ca^2+^ influx and efflux rates in mitochondria. Similar changes in histamine-induced mitochondrial Ca^2+^ were observed in LETM1-silenced B lymphocytes, hematopoietic cells, neonatal rat ventricular myocytes, and in fibroblasts from WHS patients (Doonan et al., 2014). Since the expression of MCU remained unaffected in LETM1-KD cells, the decrease in mCa^2+^ uptake rate was associated with the loss of a LETM1-dependent Ca^2+^ influx. However, the decrease in Ca^2+^ uptake rate was also accompanied by an increase in total mCa^2+^ and decrease in Ca^2+^ efflux rate. This suggested that the decreased Ca^2+^ uptake rate may be a consequence of the loss of a LETM1-dependent Ca^2+^ efflux. Thus, it is yet to be established whether LETM1-CHE facilitates Ca^2+^ influx or efflux or both (Figure 2B).

Despite studies supporting a LETM1-CHE function, recent studies have provided further evidence that human LETM1 also is primarily a KHE. Studies in HeLa cells have shown that overexpression of LETM1 increases the K^+^-stimulated proton gradient, but has no effect on the mCa^2+^ efflux rate in response to a histamine challenge (Marchi et al., 2014). Another study in HeLa cells suggested that LETM1-based regulation of mitochondrial Ca^2+^ is achieved by a LETM1-KHE activity whereby it regulates the activity of the mitochondrial Na^+^/H^+^ exchanger (mNHE), ultimately affecting NCLX-mediated Ca^2+^ efflux (Figure 2B; Austin et al., 2017). Alternatively, it may be speculated that swelling in LETM1-deficient mitochondria is caused by changes in K^+^ transport due to accumulation of Ca^2+^ (Halestrap et al., 1986; Gogvadze et al., 2004; Kaasik et al., 2007), and nigericin-mediated KHE activity may alleviate the effects of Ca^2+^ imbalance to reverse swelling in these cells. This notion is supported by the observation that nigericin stimulated a K^+^ driven H^+^ flux in LETM1-KD *Drosophila* S2 cells without restoring pH-driven Ca^2+^ exchange (Jiang et al., 2009). Thus, the controversy over the primary function of LETM1 in cells persists. In the absence of LETM1-specific inhibitors, it is even more challenging to delineate the role of LETM1 in mitochondrial Ca^2+^ regulation.

## The Diversity of LETM1-Induced Pathophysiology

### LETM1 in Cellular Metabolic Dysregulation

Evidence indicates that LETM1 mediates several steps in the cellular metabolic signaling pathway, such that LETM1 deficiency is associated with impairment of both cellular and mitochondrial metabolism. Several studies document the role of LETM1 in regulating cellular metabolism through its impact on insulin secretion and glucose uptake by cells. In mitochondria of insulin-secreting pancreatic β-cells, MCU^−/−^ as well as LETM1-KD prevented Ca^2+^-induced matrix acidification, decreased substrate-stimulated ATP production, and impaired glucose-stimulated insulin secretion (Quan et al., 2015). These observations suggest that LETM1 functions as CHE and mediates mitochondrial Ca^2+^ efflux in these cells.

However, LETM1 has also been shown to regulate cellular glucose metabolism through interactions with other signaling proteins (Figure 2A). In these examples, a LETM1-CHE/KHE function is not clear. For instance, LETM1 has been identified as a constitutive binding partner of carboxy-terminal-modulator-protein (CTMP; Piao et al., 2009b), a protein that localizes to the IMS in membrane-bound and free forms and regulates mitochondrial biogenesis by positively influencing mitochondrial fission (Parcellier et al., 2009). CTMP can bind to the hydrophobic motif of protein kinase B (Akt) and inhibit its phosphorylation (Maira et al., 2001; Parcellier et al., 2009). The nature of the interaction between LETM1 and CTMP is unknown, although, like CTMP, LETM1 has also been shown to regulate mitochondrial biogenesis. LETM1 overexpression has been correlated with increased cleavage of the mitochondrial fusion protein OPA1, leading to increased mitochondrial fragmentation (Piao et al., 2009b). It is suggested that, in obesity, LETM1 and CTMP may counter-regulate each other’s function since LETM1 expression is inversely correlated with the expression of CTMP and positively correlated with Akt phosphorylation and activity (Park et al., 2014). Thus, in liver cells from obese and high fat diet-fed mice, a decrease in LETM1 expression was associated with a reciprocal increase in CTMP expression and a decrease in Akt activity, suppression of insulin signaling and cellular glucose uptake, and development of insulin resistance (Park et al., 2014). The impact of LETM1 on insulin signaling was recently demonstrated in an epigenome-wide study of genes undergoing differential DNA methylation in obese individuals that identified one of nine differential CpG methylation sites in the LETM1 gene to be associated with fasting insulin levels (Liu et al., 2019). The association between LETM1 methylation and fasting insulin levels suggests that it can serve as an epigenetic marker to indicate future development of insulin resistance and Type II diabetes in obese individuals.

Another example of LETM1-dependent regulation of cellular metabolism, where its function as a CHE/KHE is not readily apparent is the association between LETM1 and pyruvate oxidation. Jiang et al. (2013) showed that loss of LETM1 impaired pyruvate oxidation during aerobic glycolysis, but did not affect fatty acid oxidation during fasting. This was confirmed from metabolic profiles of liver tissue, which showed significant decrease in β-hydroxybutyrate (BHB), a downstream metabolite of pyruvate, and fatty acid oxidation, in LETM1^+/−^ mice compared to WT mice during glycolysis, with no difference in BHB levels during fasting (Jiang et al., 2013). LETM1-KD also led to an increase in phosphorylated pyruvate dehydrogenase, the inactive form of the enzyme, and a decreased oxidation of pyruvate to acetyl-CoA (Durigon et al., 2018).

LETM1 also regulates mitochondrial metabolism, whereby its deficiency can decrease ATP production by compromising the assembly of respiratory complexes. A conserved 14-3-3-like domain in the LETM1 subunit has been shown to bind to the mitochondrial ribosomal protein L36 (MRPL36) and to nucleoprotein complexes called mitochondrial nucleoids, which are mitochondrial DNA (mDNA) complexed with regulatory proteins (Chen and Butow, 2005; Camara et al., 2009; Piao et al., 2009a; Lupo et al., 2011; He et al., 2012; Gilkerson et al., 2013; Durigon et al., 2018). Consequently, LETM1-KD is correlated with abnormal clustering of mitochondrial nucleoids and improper import and formation of respiratory complexes, thereby compromising mitochondrial respiration and concomitant energy metabolism (Frazier et al., 2006; Tamai et al., 2008; Lupo et al., 2011; Durigon et al., 2018). In addition, the interaction of LETM1 with BCS1L, a mitochondrial chaperone protein and AAA-ATPase, is shown to be critical for respiratory complex formation and assembly of respiratory supercomplexes. Knockdown of either LETM1 or BCS1L resulted in non-formation of complexes I and III and decreased formation of complex IV, leading to disassembly of respiratory supercomplexes, decreased ATP production, and increased formation of mitochondrial reactive oxygen species (mROS; Tamai et al., 2008). In yeast mitochondria expressing a mutant LETM1 homolog, the expression of several mitochondrially encoded proteins, including cytochrome b, a subunit of the complex III of the respiratory chain, was found to be significantly downregulated (Frazier et al., 2006). Furthermore, yeast mitochondria transfected with LETM1 lacking the MRPL36 binding domain exhibited a selective lack of respiratory complexes III and IV, suggesting a downregulation of protein translation (Lupo et al., 2011). These observations suggest that LETM1 may impact mitochondrial respiration and cellular metabolism by regulating the translation, transport, and assembly of respiratory complexes of the IMM.

In contrast, in mammalian cells, LETM1-KD was not associated with a decrease in the expression levels of respiratory chain subunits or disassembly of respiratory supercomplexes (Hasegawa and van der Bliek, 2007; Dimmer et al., 2008). Studies also showed that LETM1-KD did not affect respiratory complex assembly, but significantly inhibited complex IV activity (Doonan et al., 2014) or decreased complex II-dependent oxygen consumption (Aral et al., 2020). In both cases, mROS production was increased. The effects of LETM1-KD were partially reversed by either LETM1 reconstitution or overexpression of mitochondria-targeted expression of manganese superoxide dismutase, and glutathione peroxidase (Doonan et al., 2014) or by supplementation with melatonin, a highly effective antioxidant and free-radical scavenger (Aral et al., 2020). LETM1-deficient mitochondria exhibit a 91% increase in the frequency of “mitoflashes,” which are stochastic and discrete bursts of superoxide anion production observed in respiring mitochondria, with concomitant matrix alkalinization (Hou et al., 2014; Schwarzländer et al., 2014; Li et al., 2016; Wang et al., 2016). In these studies, the reduced mitochondrial metabolism was instead attributed to LETM1-KD-induced changes in mitochondrial morphology. This is supported by the consistent observations of disordered and swollen mitochondria in LETM1-deficient cells. Restoration of mitochondrial morphology by reconstitution of LETM1 or stimulation of K^+^ transport using ionophores suggested that the absence of a LETM1-CHE/KHE activity may underlie the distorted morphology, lack of respiratory supercomplex formation, and decreased ATP production (Nowikovsky et al., 2004; Froschauer et al., 2005; Hasegawa and van der Bliek, 2007; Dimmer et al., 2008; Hashimi et al., 2013). However, a recent study demonstrated that just the insertion of LETM1 complexes into the bilayer is sufficient to form membrane invaginations and maintain cristae structure. The structural domain in the LETM1 subunit responsible for this property lies within the conserved ribosomal binding domain (Figure 1; Nakamura et al., 2020). Considering the importance of the ribosomal binding domain for protein translation and assembly, these observations suggest that LETM1 may influence mitochondrial morphology and formation of respiratory supercomplexes independent of mitochondrial ionic homeostasis.

Thus, it is possible that different LETM1-dependent mechanisms underlie the effects of LETM1-KD on respiratory supercomplex formation and compromised bioenergetics. Further studies are required to understand how a LETM1-CHE/KHE function also regulates its interactions with proteins, such as BCS1L, MRPL36, and CTMP and impacts both mitochondrial and cellular metabolism.

### LETM1 in Neuronal Disorders

Parkinson’s disease (PD) is a progressive neurodegenerative disorder, associated with mitochondrial dysfunction (Bose and Beal, 2016). LETM1 is implicated in PD because of its putative association with the phosphatase and tensin homolog deleted on chromosome 10 (PTEN)-induced kinase 1 (PINK-1), mutations of which are found in PD models (Huang et al., 2017b). PINK-1, a mitochondria-targeted serine/threonine kinase, is imported into the IMM under normal conditions, and translocated to the OMM during mitophagy (Fallaize et al., 2015). In *in vitro* experiments, PINK-1 directly phosphorylated LETM1 at Thr192, which increased Ca^2+^ release through LETM1 in liposomes and LETM1-T192E protected PINK-1-deficient neurons by rescuing mitochondrial Ca^2+^ homeostasis (Huang et al., 2017b). The study suggests that, in neuronal mitochondria under physiological conditions, LETM1 is constitutively phosphorylated by PINK-1 and regulates Ca^2+^ handling *via* a LETM1-CHE activity (Figure 2A). Future studies elucidating the effects of PINK-1 mutations on LETM1-CHE activity will provide better insight into a possible role for LETM1 in the pathogenesis of PD.

LETM1-deficiency is linked to the pathogenesis of epileptic seizures, one of the major phenotypes in WHS patients (Endele et al., 1999; McQuibban et al., 2010; Hart et al., 2014). LETM1-deficient neurons in *D. melanogaster* exhibit motor defects, which correlates with reduced release of neurotransmitters (McQuibban et al., 2010). This is consistent with a reduced expression of LETM1 in patients with temporal lobe epilepsy and in a rat pilocarpine-induced epilepsy model, exhibiting mitochondrial swelling, early onset of the first seizure, and increased seizure frequency and duration (Zhang et al., 2014). Seizures are typically closely associated with excess ROS emission, altered metabolic pathways, and compromised mitochondrial bioenergetics and mitochondrial dysfunction (Kann et al., 2005; Folbergrová and Kunz, 2012; Khurana et al., 2013).

In patients with amyotrophic lateral sclerosis (ALS), LETM1 expression is implicated in mitochondrial dysfunction. For example, a common mouse model used to study ALS, the end-stage superoxide dismutase SOD1(G93A) transgenic mice, exhibited a 2-fold increase in LETM1 expression in motor neurons of the hypoglossal nucleus (hMNs). This correlated with a decrease in mitochondrial Ca^2+^ uptake and imbalance in mitochondrial Ca^2+^ homeostasis (Mühling et al., 2014).

The mechanism by which LETM1 expression regulates the progression of epilepsy or ALS is unknown. Nevertheless, the association of LETM1-KD-induced mitochondrial dysfunction and Ca^2+^ dysregulation suggests that the loss of a LETM1-CHE activity and resulting Ca^2+^ dysregulation may underlie pathophysiology. Interestingly, however, LETM1-KD in the epilepsy model also decreased the expression of mitochondrial cytochrome b, a component of complex III (Zhang et al., 2014). Given the role of LETM1 in the formation of respiratory supercomplexes and protein import *via* interactions with BCS1L and MRPL36, it is likely that this aspect of LETM1-depedent regulation also plays a role in the effects of LETM1-KD in epilepsy. Furthermore, LETM1^+/−^ mice manifest both impaired glucose metabolism and seizure activity (Jiang et al., 2013). Given that LETM1 regulates mitochondrial and cellular metabolism (described in “LETM1 in cellular metabolic dysregulation” in this article), this indicates that LETM1-deficiency induced mitochondrial dysfunction underlies both PD and ALS.

### LETM1 in Cancer

Cancer cells depend upon mitochondria for metabolic reprogramming in cancer initiation, progression, and resistance to therapy (Vyas et al., 2016). LETM1-mediated regulation of mitochondrial biogenesis, metabolism, and cell survival signaling has provided impetus to investigate LETM1 activity in various human cancers (Dimmer et al., 2008; Piao et al., 2009a; Li et al., 2015). Multiple human malignancies, including breast, colon, esophagus, lung, ovary, rectum, stomach, and uterine cervix, report high LETM1-expression profile correlating with poor prognosis, low survival rates, upregulation of cancer stemness genes, and enhanced angiogenesis (Piao et al., 2009a, 2019a,b, 2020; Li et al., 2015, 2020; Wang et al., 2015, 2020; Huang et al., 2017a; Yang et al., 2018). In thyroid, prostate, ovarian, and gastric cancers, LETM1 overexpression is correlated with increased cell survival *via* enhanced Akt signaling (Lee et al., 2016; Piao et al., 2020; Wang et al., 2020; Zhang et al., 2020). This is further corroborated by the observation in prostate cancer cells, that the PI3K inhibitor LY294002, which stopped the migration and invasion of tumor cells, also caused the downregulation of LETM1 (Piao et al., 2020). This suggests that LETM1 may mediate Akt signaling through its interaction with the Akt inhibitor, CTMP. In lung cancer, LETM1 overexpression promoted tumor formation by inhibiting 5'-adenosine monophosphate activated protein kinase (AMPK), a cellular bioenergetic sensor that activates autophagy at depleted ATP levels (Hwang et al., 2010). AMPK inhibition would lead to deregulation of the normal progression of the cell cycle. Accordingly, LETM1-KD reduced the expression of cancer stemness-related genes, increased AMPK activation, arrested cell cycle, lowered the number and size of tumor spheroids, and markedly inhibited cell proliferation (Che et al., 2020; Li et al., 2020; Piao et al., 2020).

In contrast with studies that associate LETM1-overexpression with promotion of tumorigenesis, other studies demonstrated that LETM1 over-expression impaired mitochondrial biogenesis, compromised ATP production, and elicited necrotic cancer cell death in lung (Hwang et al., 2010) and hepatocellular carcinoma (Shin et al., 2013). In these studies, LETM1 overexpression correlated with suppression of Akt activity and cell survival signaling while facilitating apoptosis (Hwang et al., 2010; Shin et al., 2013). However, it is to be noted that in Shin et al. (2013), co-delivery of LETM1 with CTMP to tumor cells as a treatment strategy in hepatocellular carcinoma, led to overexpression of both LETM1 and CTMP and correlated with distorted mitochondrial morphology and increased apoptosis. The authors surmised that the decrease in Akt phosphorylation was a result of increased apoptosis caused by mitochondrial dysfunction in LETM1-overexpressed cells.

Alterations in cellular and mitochondrial Ca^2+^ homeostasis and ensuing mitochondrial dysfunction are a hallmark of tumorigenesis (Romero-Garcia and Prado-Garcia, 2019). It remains to be determined how changes in LETM1 expression alter mitochondrial Ca^2+^ regulation. The correlation of LETM1 expression with Akt phosphorylation suggests that, in addition to LETM1-CHE/KHE activity, regulation of tumorigenesis by LETM1 may involve signaling through its constitutive binding partner CTMP in cancer cells.

LETM1 may also be involved in initiating resveratrol-induced cancer cell death (Madreiter-Sokolowski et al., 2016). In this study, resveratrol, a naturally occurring polyphenol, induced apoptosis in HeLa and human vascular endothelial (EA.hy926) cancer cells but not somatic cells. The authors proposed that the specific inhibitory action of resveratrol on the F1 subunit of ATP synthase may decrease ATP production and inhibit the activity of SERCA in the MAM region of cancer cells, triggering SOCE in these cells (Madreiter-Sokolowski et al., 2016). In the absence of SERCA-mediated Ca^2+^ sequestration in the ER, this would lead to increased MCU/LETM1-mediated Ca^2+^ uptake into mitochondria, resulting in mitochondrial Ca^2+^ overload and apoptosis (Waldeck-Weiermair et al., 2013). This hypothesis provides a possible mechanism by which the increased expression of LETM1 in cancer cells may be utilized as a means for delivering therapy. However, this hypothesis remains to be investigated. A comprehensive understanding of the mechanism by which LETM1 regulates mitochondrial Ca^2+^ handling, interactions with regulatory proteins such as CTMP and inter-organelle signaling is necessary to explore LETM1 as a potential reliable prognosticator and therapeutic target in cancer treatment.

## Perspective

The diverse impact of LETM1 deficiency, as outlined above, suggests that LETM1 expression and function is critical for short-term dynamic processes such as cellular metabolism as well as long-term processes important for cell survival and development. Although the primary function of LETM1 as CHE or KHE is still being debated, it is evident that loss of LETM1 function adversely impacts mitochondrial Ca^2+^ handling in several cell types. Whether, this is due to the loss of a LETM1-CHE function or a consequence of the loss of a LETM1-KHE function on mitochondrial Ca^2+^ handling needs to be resolved. The association between changes in LETM1 expression and effects on mitochondrial Ca^2+^ handling as described in this review argues in favor of a CHE function for LETM1. It is to be noted, however, that evidence for LETM1-CHE activity has been largely obtained from observations in *in vitro* experimental conditions where the LETM1 protein is studied in isolation, devoid of other ionic activities that determine ionic homeostasis, and osmotic balance *in vivo* (Jiang et al., 2009; Tsai et al., 2014; Shao et al., 2016). Conversely, cell studies covering the spectrum from lower to higher-order eukaryotes, while providing compelling evidence for an evolutionarily conserved LETM1-KHE function (Nowikovsky et al., 2004; Dimmer et al., 2008; McQuibban et al., 2010; Hashimi et al., 2013), do not account for the role of EF-hands in mammalian LETM1 in a KHE function. Our recent study in rat cardiac mitochondria conducted in Na^+^-free conditions to render NCLX inactive, showed that the contribution of a Na^+^-independent Ca^2+^ efflux (CHE), presumably encoded by LETM1, increased with matrix Ca^2+^ load and that CHE may be able to sense matrix Ca^2+^ buffering (Natarajan et al., 2020). The studies favoring a LETM1-KHE function cannot explain how a LETM1-KHE may regulate Ca^2+^ handling in the absence of NCLX. Future studies will need to address these knowledge gaps as to how the LETM1-CHE function in *in vitro* conditions is modulated in the presence or absence of one or more EF-hands in the LETM1 protein or factors such as the presence of other ionic exchangers.

LETM1 interacts with proteins, such as CTMP, BCS1L, and MRPL36, and regulates processes like Akt signaling and protein import and assembly. Changes in LETM1 expression also affect these processes, implicating it in the pathophysiology of several disease phenotypes, including metabolic, neurodegenerative, and neoplastic diseases (Figure 2C). The link between the primary function of LETM1 as CHE/KHE and its interaction with these proteins is not apparent. Specifically, is the CHE or KHE function of LETM1 independent of its actions on signaling proteins and respiratory complex assembly? Some insight is obtained from a recent study, which showed in *in vitro* experiments that insertion of LETM1 complexes into membrane bilayers alone led to the formation of membrane invaginations (Nakamura et al., 2020). Four residues in the C-terminus of the LETM1 subunit were found to be critical for LETM1 complex formation and maintaining morphology of the IMM (Nakamura et al., 2020), which is crucial for the proper assembly and function of essential IMM proteins (Bulthuis et al., 2018). These residues are distinct from the transmembrane and EF-Hand domains of the LETM1 subunit, which are important for LETM1-CHE activity. Since the formation of LETM1 complexes is regulated by the chaperone protein BCS1L (Tamai et al., 2008), this suggests that the interaction of LETM1 with BCS1L may occur at sites independent of the domains that regulate the LETM1-CHE/KHE function. It is, however, possible that the domain(s) regulating LETM1-CHE/KHE would influence the interaction of LETM1 with proteins such as BCS1L and hence the regulation of mitochondrial morphology and vice versa. Given the increasing importance of inter-organelle communications and signaling for integrated cellular function in health and disease (Javadov et al., 2020), this aspect of LETM1-mediated signaling, in addition to its exchanger function needs further investigation.

## Author Contributions

All authors listed have made a substantial, direct and intellectual contribution to the work, and approved it for publication.

### Conflict of Interest

The authors declare that the research was conducted in the absence of any commercial or financial relationships that could be construed as a potential conflict of interest.
